# Nursing Homes and COVID-19: Staff Experiences

**DOI:** 10.1093/geroni/igab046.954

**Published:** 2021-12-17

**Authors:** Verena Cimarolli, Joann Reinhardt, Sheryl Zimmerman

## Abstract

Nursing homes (NHs) faced an unprecedented crisis during the rapid spread of COVID-19. This pandemic has had a devastating impact on both NH residents and workers who are often on the frontlines providing hands-on care. These workers are vulnerable to the health risks of COVID-19 due to daily exposure to residents with COVID-19, residence in areas with high infection rates, and challenges specific to low-income workers (e.g. reliance on mass transportation). Research has highlighted the experiences of NH workers during the pandemic to learn how to better support them now and during future pandemics. This symposium will add to this research and present new findings from studies conducted in the United States to capture the unique experiences of NH employees. First, Bryant illustrates specific COVID-19-related challenges that NH frontline workers faced and how these workers’ experiences compare to workers in other long-term services and support settings. Reinhardt reports findings from a qualitative study examining the multi-level challenges experienced by nursing assistants during the pandemic. Cimarolli examines if quality of employer communication and workers’ perceived COVID-19-related preparedness mitigate the impact of work-related stress on NH workers’ decision to resign. Franzosa shares recommendations based on priorities identified by nursing assistants and administrators to build future resilience based on lessons learned. Finally, Simpson identifies factors associated with states’ decisions to adopt COVID-19 testing mandates for workers in NHs. Dr. Zimmerman discusses study findings and their contributions for creating supportive NH work environments to ensure most optimal NH worker and resident quality of life.

